# The validity of the SF-36 in an Australian National Household Survey: demonstrating the applicability of the Household Income and Labour Dynamics in Australia (HILDA) Survey to examination of health inequalities 

**DOI:** 10.1186/1471-2458-4-44

**Published:** 2004-10-07

**Authors:** Peter Butterworth, Timothy Crosier

**Affiliations:** 1 Centre for Mental Health Research, The Australian National University, ACT 0200 Australia; 2 Strategic Policy and Knowledge Branch, Department of Family and Community Services, Canberra, Australia

## Abstract

**Background:**

The SF-36 is one of the most widely used self-completion measures of health status. The inclusion of the SF-36 in the first Australian national household panel survey, the Household, Income and Labour Dynamics in Australia (HILDA) Survey, provides an opportunity to investigate health inequalities. In this analysis we establish the psychometric properties and criterion validity of the SF-36 HILDA Survey data and examine scale profiles across a range of measures of socio-economic circumstance.

**Methods:**

Data from 13,055 respondents who completed the first wave of the HILDA Survey were analysed to determine the psychometric properties of the SF-36 and the relationship of the SF-36 scales to other measures of health, disability, social functioning and demographic characteristics.

**Results:**

Results of principle components analysis were similar to previous Australian and international reports. Survey scales demonstrated convergent and divergent validity, and different markers of social status demonstrated unique patterns of outcomes across the scales.

**Conclusion:**

Results demonstrated the validity of the SF-36 data collected during the first wave of the HILDA Survey and support its use in research examining health inequalities and population health characteristics in Australia.

## Background

While much health research focuses on objective outcome measures such as mortality or morbidity defined through clinical assessment, there is an increasing emphasis on self-reported measures of health status and health-related quality of life. Self-reported measures of health status have been included in epidemiological and community-based survey research. Their use reflects the importance of considering the patients' point of view and the multidimensional nature of health [1-3].

The Medical Outcomes Study Short Form (SF-36) is one of the most widely used, self-completion measures of health status. It was developed to meet the psychometric standards necessary for group comparisons, to enable profiling of functional health and well-being, and to quantify disease burden [3]. It comprises 36 items of which all but one are used to measure eight important health concepts that are frequently examined through health surveys. These eight concepts or scales are: Physical Functioning; Role-Physical (interference with work or other daily activities due to physical health); Bodily Pain; General Health; Vitality; Social Functioning (interference with normal social activities); Role-Emotional (interference with work or other daily activities due to emotional problems); and Mental Health (symptoms associated with anxiety and depression and measures of positive affect). In addition, the eight scales yield two summary scales of health, relating to physical (the Physical Component Summary: PCS) and mental (the Mental Component Summary: MCS) functioning and well-being.

The SF-36 was first adapted for use in Australia in 1992, as part of the International Quality of Life Assessment (IQOLA) Project [4]. Previous research has demonstrated the validity of the SF-36 for use by Australian respondents using samples from Canberra and New South Wales [2,4]. This has involved assessment of the psychometric properties of the Australian form of the SF-36, evaluation of internal consistency and reliability, and demonstration of content and construct validity. There are considerable Australian data on the SF-36 from large National samples. In 1995, a subset of National Health Survey respondents (around 18,800 adults) completed the SF-36 and the Australian Bureau of Statistics published Australian population norms [5]. The SF-36 was also included in the Women's Health Australia survey, with data collected from a sample of around 41,500 women aged 18–22, 45–49, and 70–74 [6].

In 2001, the SF-36 was included in the first wave of the Household, Income and Labour Dynamics in Australia (HILDA) Survey. This is an Australia-wide survey of approximately 7,680 households, comprising around 14,000 people aged 15 and over. The HILDA Survey is the first longitudinal household survey in Australia and is designed to provide a sound evidence base to support research and analysis of income, labour market and family dynamics. As such, it is a critical resource for social policy development. The inclusion of the SF-36 in the HILDA Survey will enable investigation of the interaction between social, economic and health measures.

There is an extensive body of research demonstrating socio-economic inequalities in the distribution of physical and mental health problems [7-9]. The SF-36 has been utilised in research on health inequalities and this research has shown that the SF-36 scales are differentially associated with markers of social-economic circumstance [10-13]. A focus of our research has been on the nature of disadvantage and social exclusion associated with welfare receipt and specifically the association between welfare receipt and mental health problems [14]. The HILDA Survey provides a valuable dataset with which to investigate this research topic. As such, it is critical to firstly ascertain the validity of the data within the HILDA Survey. Further, demonstrating the validity of the SF-36 scales collected through the survey is critical for other researchers and policy analysts who will utilise the HILDA dataset.

The aim of this paper is to evaluate the psychometric properties and criterion validity of the HILDA SF-36 data. We have used the manuscript by Sanson-Fisher and Perkins [4] as a framework for the analyses reported in the first section. This follows the standard IQOLA validation procedures [15]. The analysis examines the reliability and validity of the eight SF-36 scales and the PCS and MCS scales. We then compare the SF-36 results obtained from the HILDA Survey with other Australian estimates to assess the representativeness of the data. We also evaluate the criterion validity of the HILDA Survey SF-36 data. We do this by 1) looking for convergent validity, in which scales measuring similar or related constructs demonstrate a positive association, and divergent validity whereby unrelated scales and measures are not associated; and 2) examining the profile of SF-36 results across a range of measures associated with health, disability and social functioning, demographic characteristics as well as a focus on a number of measures of socio-economic circumstances.

## Methods

### Data source

Data are from the first wave of the HILDA Survey (Release 1.0), a nationally representative household panel survey. The HILDA Survey was funded by the Australian Department of Family and Community Services and managed by a consortium led by the Melbourne Institute of Applied Economic and Social Research at the University of Melbourne. The survey utilised a multi-stage sampling approach (sampling households within Census Collection Districts) and was stratified by State and part-of-State.

Four survey instruments were included in Wave 1. A *Household Form *and *Household Questionnaire *were completed during a personal interview with one adult member of each household. The *Person Questionnaire*, also administered during the personal interview, was conducted with all adult household members. Finally, the *Self-Completion Questionnaire *(SCQ) was provided to all respondents to the Person Questionnaire and was collected at a later date or returned by post. The SF-36 was included as the first element in the SCQ. Fieldwork for wave one of the HILDA Survey was conducted between August 2001 and January 2002.

A total of 7,682 households responded to the survey (a household response rate of 66 per cent). Within these households, there were 15,127 eligible adults. Of this group 13,969 (92%) completed the Person Questionnaire and 13,055 (86%) completed and returned an identifiable SCQ.

There are some differences between the characteristics of respondents to the HILDA Survey and population estimates from the Australian Bureau of Statistics. However, these discrepancies are not large enough to discredit the data and the differences in rates of response across both sex and location are corrected by applying population weights [16].

### Analysis

The first set of analyses evaluated the reliability and validity of the SF-36 scales in the HILDA Survey. Given that these analyses were concerned with the internal structure of the data rather than representativeness, we ignored the clustered and stratified nature of the data and did not take population weights into account. These analyses were conducted using SPSS version 11.7.

Item-internal consistency assessed the extent to which each item measures what its associated scale measures. A correlation of 0.40 (corrected for overlap) or greater demonstrates adequate item-internal consistency [17]. Item-discriminant validity was also assessed. This is demonstrated when an item correlates significantly more strongly with the scale it contributes to rather than with any other scale. We use the multitrait/multi-item correlation matrix approach in which the correlation of each item with each scale is examined [18]. In this approach, the correlation between each item and its own scale is corrected for overlap: that is, the scale is calculated without the specific item in question to avoid inflating the correlation. The extent to which item-scale correlations within a scale were equal was also assessed, as was the approximate equality of item means and standard deviations.

To assess the internal consistency of scale scores, Cronbach's alpha coefficients were assessed, with a criterion of 0.7 used to define adequate internal consistency. Descriptive statistics for the eight SF-36 scales and the two summary scales were calculated, including the mean, median, range, standard deviation, skewness, kurtosis, and the per cent scoring at the lowest value (floor) and the highest value (ceiling).

Construct validity of the SF-36 in the HILDA Survey was also evaluated using the principal components method of factor analysis. Results were compared with the factor structure obtained from other analysis of the SF-36 scales.

The second set of analyses also examined a form of construct validity, assessing the extent to which scores on the SF-36 scales were associated with other criteria. We included in our analysis a number of measures of health and disability, ratings of satisfaction with life and health, and measures of stressful employment conditions and persistent feelings of loneliness. In addition to examining the profiles across the eight SF-36 scales, we also examined the relationship between these criterion measures and the PCS scale and the MCS scale, which were calculated according to the standard procedure outlined by Ware, Kosinski and Keller [19].

For these analyses it was critical to take account of the complex survey design. We therefore utilised the *svy *procedures of STATA to account for the clustered and stratified nature of the data, and to facilitate the use of weights to overcome differential response rates and to replicate Australian population parameters. The HILDA Survey dataset contains person-level weights which, when applied to individual survey respondents, adjusts for the unequal probabilities of selection and completion of the Person Questionnaire. However, these weights do not adjust for the further attrition associated with the SCQ. This is critical as our analysis is focused upon measures from this questionnaire. We therefore, conducted a logistic regression analysis to predict completion of the SCQ using the same predictors used to derive the HILDA person-level weights (geographic location, labour-force status, sex, age, number of adults in household, number of children in household, marital status, English language ability, and dwelling type) [20]. The probabilities of responding derived from this analysis were used to adjust the person-level weights. Utilising these adjusted weights produced accurate population estimates from the respondents who completed the SCQ (similar to the estimates for Personal Questionnaire respondents using the original person-level weights).

We also examine the correspondence between SF-36 results from the HILDA Survey and data from another, large-scale Australian survey to assess the representativeness of the data. We contrast the means for each of the SF-36 scales from the HILDA Survey with those obtained by the Australian Bureau of Statistics through the National Health Survey conducted in 1995 [5]. To facilitate evaluation of potential differences between means, two sample t-test statistics were calculated using a pooled variance approach.

### Demographic, socio-economic and health measures

Based on previous analysis of the SF-36, we expected results across the eight scales to differ according to subgroups identified by demographic and socio-economic characteristics, including age, sex, marital status, educational attainment, housing tenure and employment status. We also examined differences associated with receipt of welfare payments, a focus of our research endeavours [14]. The analyses also include a range of measures related to health and life circumstances drawn from other scales and instruments included in the HILDA Survey.

#### Long-term health condition

One question from the Person Questionnaire asked respondents if they experienced a disability or health condition that had lasted, or was likely to last 6 months or more, and restricted their everyday activities. A showcard listing examples of health conditions, impairments and disabilities was presented as a prompt for respondents. Responses were either *Yes *or *No*.

#### Limited ability to work

Those identifying a disability or condition were asked to rate whether this limited the type or amount of work they were able to undertake. Response categories were *No*, *Yes *and *Can Do Nothing*.

#### Satisfaction with health

In a series of questions from the Person Questionnaire, respondents were asked to rate their satisfaction with a range of life circumstances using an eleven-point scale with descriptive anchors at either end (0 = totally dissatisfied; 10 = totally satisfied). Responses were categorised as either dissatisfied (if the rating was 4 or less) or satisfied/ mixed (if the rating given was at the midpoint 5 or higher).

#### Satisfaction with life

Respondents were also asked, all things considered, how satisfied they were with their life. Responses were categorised as above.

#### Job stress

The SCQ included a series of statements about current job characteristics, to which respondents rated their level of agreement or disagreement using a seven-point scale anchored at either end (1 = strongly disagree; 7 = strongly agree). One item asked respondents to rate whether they feared that the amount of job stress would make them physically ill. Respondents were categorised as experiencing significant job stress if they agreed with this statement (a rating of 5 or greater).

#### Social isolation

The final measure was also drawn from the SCQ. Using the same scale as the job stress measure, respondents were presented with a series of statements about their level of social support. Respondents were categorised as lonely if they agreed (rating of 5 or above) with the statement "*I often feel very lonely*".

Based on previous research using the SF-36, we anticipate that increasing age will be associated with poorer physical health, and that women will demonstrate poorer health than men as measured on all SF-36 scales with the possible exception of the General Health scale [5]. Those respondents who are divorced or separated are expected to demonstrate poorer health than those currently married or in a de facto relationship, with the greatest differences observed on the scales related to social functioning and mental health [21]. For all of the socio-economic measures, we expect to find a socio-economic gradient for all SF-36 scales, though the pattern may differ for different measures. For example, Sullivan and Karlsson found that educational level was more strongly associated with the measures of physical health whereas employment status was more strongly associated with mental health. Also based on results such as those reported by Sullivan and Karlsson, it was expected that reported experience of a long-term health condition, limited ability to work and satisfaction with health would be more strongly associated with SF-36 scales related to physical health, while job stress, social isolation (loneliness) and satisfaction with life would be more strongly associated with the scales loading on the mental health factor.

## Results and discussion

### Sample statistics

Of the 13,055 respondents with identifiable SCQs, 53 percent were female. The age of respondents ranged from 15 to age 90 or greater (reported age was capped at 90 in the survey data), with an average age of 43 years (SD = 17.25).

A total of 11,264 respondents (86.3%) completed all SF-36 items, with a further 1,326 (10.1%) having 5 or fewer missing items. Overall, the mean number of missing items per person was 0.69, and the median was 0. Details of non-responses/missing values for each item are presented in Figure A1 of the Attached file. The rate of missing values was below 3.5 percent for all items. The highest rate of missing values was evident for items from the Physical Functioning scale, followed by the Role Physical, General Health and Role Emotional scales. Scales were calculated using standard SF-36 scoring procedures, whereby missing values were replaced by scale means where valid responses were available for at least half of the scale items. Therefore, the number of respondents with valid scale scores ranged from 12,686 for the Role Emotional scale to 13,031 for the Social Functioning scale. Analyses were conducted with the maximum number of respondents possible and, therefore, varies across the scales.

### Tests of scaling assumptions

Table 1 demonstrates that the range of item-scale correlations within each scale were moderate to strong (see item-internal consistency column). Indeed, all item-scale correlations were greater than the recommended correlation of 0.40 for adequate item-internal consistency [17]. The widest range of item-scale correlations was observed for the General Health scale (0.47 – 0.77), with the weakest item-scale correlations evident for items 11a and 11c (see Table A1 in Additional file). Nonetheless, the item-scale correlations were reasonably similar within each scale.

**Table 1:** Results of item scaling tests and reliability estimates for SF-36 scales.

		**Range of item correlations**		
	**No. of items**	**Item-internal consistency**^1^	**Item-discriminant validity**^2^	**Scale Reliability**^3^
**SF-36 scale**				
Physical Functioning	10	0.55 – 0.81	0.14 – 0.55	0.93
Role Physical	4	0.76 – 0.81	0.24 – 0.60	0.90
Bodily Pain	2	0.80	0.32 – 0.67	0.87
General Health	5	0.47 – 0.77	0.22 – 0.57	0.82
Vitality	4	0.63 – 0.67	0.24 – 0.59	0.83
Social Functioning	2	0.71	0.42 – 0.60	0.83
Role Emotional	3	0.68 – 0.72	0.27 – 0.54	0.83
Mental Health	5	0.54 – 0.69	0.15 – 0.57	0.82

^1 ^Correlations between items and scale corrected for overlap.

^2 ^Correlations between items and other scales.

^3 ^Internal-consistency reliability (cronbach's alpha).

In order to assess item-discriminant validity, items that make up a scale were correlated with other scale constructs. Item-discriminant validity is demonstrated when an item correlates higher with its own scale than with other scales. While there was some overlap in correlations between items from one scale and other scales (compare the range of item correlations for the item-internal consistency and item-discriminant validity columns in Table 1), these were relatively minor. At the level of the individual items, it is apparent that all items were more strongly correlated with their own scale than with other scales, and that this difference was statistically significant for all but one item (see Table A.2 in Additional file). Thus, Table A.3 demonstrates the achievement of 100% scaling success according to accepted SF-36 methods.

### Reliability

Table 1 also shows that all SF-36 scales demonstrated acceptable internal consistency, with Cronbach's alpha ranging from 0.82 (Mental Health and General Health) to 0.93 (Physical Functioning). These reliability scores are similar to those reported in previous Australian research [2,4].

### Descriptive statistics for scales

In accordance with the standard scoring procedures [19], the eight scales of the SF-36 were constructed by aggregating 35 of the 36 individual items (the excluded item measures self-reported health transition). Each of the 35 items contributed to one scale, with each scale comprising 2 to 10 items. The range of scores possible on each of the eight scales was from 0 to 100, with 100 representing optimal functioning as measured by the SF-36.

The (unweighted) means for the eight scales ranged from 60.87 (Vitality) to 83.19 (Physical Functioning; see Table 2). All scales were found to be negatively skewed with the Physical Functioning, Role Emotional, Role Physical and Social Functioning scales moderately skewed. Ceiling effects were high for the Role Emotional (73.3%) and Role Physical (68.8%) scales and moderate for the Social Functioning (49.4%), Physical Functioning (33.8%) and Bodily Pain (32.9%) scales. The greatest prevalence of floor effects was observed for the Role Physical (12.4%) and Role Emotional (9.9%) scales. Minimal ceiling and floor effects were observed for the Vitality, Mental Health and General Health scales. This pattern is similar to that obtained with previous assessment of the SF-36 [4]. Descriptive statistics for the two summary scores (the Physical and Mental Component Summary scores) are also consistent with previous findings.

**Table 2:** Descriptive statistics for SF-36 scales.

**SF-36 scale**^1^	**No. of items**	**Mean**	**Median**	**Range**	**SD**	**Skewness**	**Kurtosis**	**Floor (%)**	**Ceiling (%)**
PF	10	83.19	95.00	0–100	23.11	-1.70	2.10	0.5	33.8
RP	4	79.11	100.00	0–100	35.58	-1.42	0.40	12.4	68.8
BP	2	74.30	84.00	0–100	25.19	-0.81	-0.20	0.9	32.9
GH	5	70.02	72.00	0–100	21.14	-0.80	0.16	0.2	6.1
VT	4	60.87	65.00	0–100	19.84	-0.59	-0.01	0.4	1.1
SF	2	81.96	87.50	0–100	23.55	-1.29	0.86	0.6	49.4
RE	3	82.13	100.00	0–100	33.08	-1.64	1.16	9.9	73.3
MH	5	73.92	76.00	0–100	17.42	-0.98	0.88	0.1	3.3
PCS	21	49.17	52.55	3.61–71.87	10.33	-1.19	0.86	0.0	0.0
MCS	14	49.92	52.74	4.45–73.94	9.95	-1.18	1.24	0.0	0.0

^1 ^PF = Physical Functioning; RP = Role Physical; BP = Bodily Pain; GH = General Health; VT = Vitality; SF = Social Functioning; RE = Role Emotional; MH = Mental Health; PCS = Physical Component Summary; MCS = Mental Component Summary.

### Principal Components Analysis

Principal Components Analysis was conducted to examine the underlying structure in the SF-36. The analysis supported the two factor solution. Two factors had eigenvalues greater than one. The two factor solution was also consistent with the pattern of results evident in the scree plot. The two factors accounted for 69% of the total variance in the 8 SF-36 scales. The first factor accounted for 56% of the variance and the second factor accounted for an additional 13%. The total variance accounted for by the two-factor solution was similar to that found in other studies. For example, in a ten country comparison of the factor structure of the SF-36, Ware and colleagues report that the two factor solution accounted for between 66 and 72 percent of total variance [22]. Previous Australian analyses also found that the two factors explained around 70 percent of total variance [4,6].

Table 3 shows the correlations between the SF-36 scales and the rotated components. We used the varimax method to obtain orthogonal factors. Across the eight scales, the percentage of variance within each scale explained by the two-factor solution (commonalities) ranged from 0.57 to 0.82. Again, this is consistent with previous international studies [22]. It is apparent from Table 3 that our two-factor solution is also consistent with the previously reported factor structure, identifying scales that relate to physical and mental health. The scales that correlated strongly with the first factor were Mental Health (0.90), Vitality (0.74), Role Emotional (0.71) and Social Functioning (0.71). The Physical Functioning (0.11), Role Physical (0.28) and Bodily Pain (0.31) scales correlated weakly with this factor. This factor was subsequently labelled mental health. With regard to the second factor, the Physical Functioning (0.84), Role Physical (0.81) and Bodily Pain (0.77) scales correlated strongly, while the Mental Health (0.08) and Role Emotional (0.25) scales correlated weakly. This factor was labelled physical health.

**Table 3:** Rotated principal components associations between the eight SF-36 scales and the mental and physical health components. Commonalities (h_2_) are also presented.

	Rotated principal components		
		
**SF-36 scale**	**Mental**	**Physical**	**h**^2^
Physical Functioning	.106	.842	0.72
Role Physical	.278	.806	0.73
Bodily Pain	.307	.771	0.69
General Health	.475	.627	0.62
Vitality	.744	.361	0.68
Social Functioning	.709	.465	0.72
Role Emotional	.714	.246	0.57
Mental Health	.900	.079	0.82

As with most international comparisons (though see [23,24] using Taiwanese and Japanese populations), the factor loadings associated with General Health were stronger for the physical health factor. We also found this pattern of results, though the relationship observed between the General Health scale and the mental health factor was at the upper end of the range of international data. The opposite pattern is generally demonstrated for the Vitality scale, with it loading most strongly on the mental health factor. One previous Australian study [4] found that the Vitality scale loaded more strongly on the physical health factor. The current results are more consistent with the body of international evidence.

### Normative comparisons

Table 4 presents population estimates of the mean and standard deviation for each of the SF-36 scales and the Physical and Mental Component Summary scores. These results take account of the complex survey design and population weights. Also presented are the corresponding results from the ABS 1995 Health Survey.

**Table 4:** Population estimates of mean SF-36 Scale scores and Component Summary Scores (with N and standard deviation) from the HILDA Survey. Means (and sd) from ABS (1995) are also presented, together with estimate of independent t test to assess differences between estimates.

	**HILDA Population **(weighted)		**Australian population norms**^1^		
	Mean (sd)	N	Mean (sd)	N	*t test*
**Physical Component Summary Score **^2^	**49.23 **(10.6)	**12 320**			
**Mental Component Summary Score **^2^	**49.79 **(10.3)	**12 320**			
**Scales**					
**Physical Functioning**	82.49 (24.0)	12 760	82.6 (23.9)	18 734	0.40
**Role Physical**	79.06 (36.2)	12 714	79.9 (35.1)	18 710	2.06
**Bodily Pain**	73.89 (25.6)	12 977	76.8 (25.0)	18 699	10.09
**General Health**	69.54 (21.6)	12 718	71.6 (20.3)	18 715	8.60
**Vitality**	60.72 (20.0)	12 938	64.5 (19.8)	18 728	16.64
**Social Functioning**	81.14 (24.0)	13 031	85.0 (22.5)	18 789	14.64
**Role Emotional**	81.83 (33.8)	12 686	82.9 (32.3)	18 620	2.82
**Mental Health**	73.42 (17.7)	12 930	75.9 (17.0)	18 676	12.55

^1 ^From ABS, 1995

^2 ^Physical and mental summary scores were calculated differently in the ABS publication (using factor scores from principle component analysis and stardardised to have a mean of 50 and sd of 10). Therefore, it is not appropriate to directly compare with the HILDA data derived using the standard scoring procedures.

The results obtained are broadly consistent with the previous Australian national data. The means are of a similar magnitude. Direct comparison of the means, assessed using independent t tests (see [6]), indicated that the differences observed were statistically significant (at the .01 level) for six of the eight scales. The mean scores from the HILDA Survey were consistently lower than those obtained in the ABS Health Survey. However, the greatest differences observed on the individual scales (VT and SF) were less than four points and Ware et al. [19] suggest that a difference of 5 points or more indicates clinical or social meaningfulness.

While relatively small, the significant differences in mean SF-36 scale scores from these two national surveys are intriguing. The most marked differences were on scales related to mental health, while the scales most strongly related to physical health (the Physical Functioning and Role Physical scales) were those where no difference between surveys was observed. It may be that the differences observed reflect different methodological approaches adopted in the two surveys, or could be due to non-sampling error. Alternatively, it could reflect a real change in the Australian population between 1995 and 2001, with poorer reported mental health occurring in more recent times. This may be the case, as changes were restricted to the measures related to mental health and therefore are not simply a general change in response bias.

There is evidence which corroborates the apparent increased prevalence of mental health problems in the community. Comparison of the 1997 Australian Survey of Mental Health and Wellbeing and the 2001 National Health Survey, both of which included the 10 item Kessler Psychological Distress Scale, showed the rates of substantial psychological distress had increased by over one percent [25]. In the UK, comparison of the results from the 1993 and 2000 Psychiatric Morbidity Surveys found that, while there was no difference in the overall rate of neurotic disorders amongst adults, there was a significant increase in the prevalence among men [26]. Further investigation of this finding and possible explanations (e.g. increasing mental disorders, reduced stigma and increased likelihood of disclosure) is warranted.

### Comparison of groups

The data presented in Table 5 show the profile of mean SF-36 scores across a range of demographic and socio-economic characteristics. There were small but significant sex differences across most of the SF-36 scales and summary measures. Females consistently demonstrated slightly poorer health than males on all measures apart from the General Health scale, including the physical and mental summary scores. This pattern of results precisely replicates the pattern found in the previous ABS National Health Survey [5].

**Table 5:** Mean SF-36 Scale and Component Summary Scores, by measures of demographic and socio-economic circumstances. The *F *statistic associated with each comparison is indicated, together with minimum number of respondents in each cell (in brackets).

	**PCS**	**MCS**	**PF**	**RP**	**BP**	**GH**	**VT**	**SF**	**RE**	**MH**
**Gender**										
Male (5817)	49.49	50.45	83.84	80.23	74.96	69.51	62.63	82.21	83.46	74.61
Female (6503)	48.97	49.15	81.18	77.90	72.84	69.57	58.85	80.09	80.22	72.25
*F(1,475)*	*8.19*	*53.82*	*44.89*	*14.56*	*24.03*	*0.03*	*121.39*	*27.94*	*30.55*	*62.19*
	**	***	***	***	***	*ns*	***	***	***	***
**Age**										
18–25 (2135)	53.23	48.26	91.75	88.48	80.26	73.38	62.53	82.65	83.09	71.64
26–39 (3535)	52.16	48.88	89.17	86.40	78.71	73.85	60.97	82.50	83.91	72.80
40–55 (3676)	49.56	49.99	84.28	80.82	73.70	69.49	60.86	81.71	83.35	73.74
56–65 (1452)	44.47	51.51	72.87	69.27	66.23	63.72	60.87	79.87	80.42	74.44
66–75 (992)	41.70	52.92	65.10	61.29	64.59	62.26	59.47	79.61	78.90	76.14
75+ (530)	35.85	51.31	49.49	39.71	57.64	55.57	53.21	70.22	62.76	75.01
*F(5,451)*	*324.72*	*29.94*	*302.04*	*210.84*	*96.79*	*85.05*	*15.69*	*17.57*	*25.92*	*8.15*
	***	***	***	***	***	***	***	***	***	***
**Marital status**										
Married/de facto (7928)	48.85	50.73	82.36	79.15	73.68	69.62	61.08	82.97	83.74	74.93
Divorced/separated (1013)	47.01	47.50	77.23	71.74	67.25	65.87	57.02	72.82	73.94	69.29
*F(1.475)*	*18.74*	*59.43*	*29.33*	*28.06*	*31.20*	*19.39*	*25.41*	*94.83*	*52.03*	*60.11*
	***	***	***	***	***	***	***	***	***	***
**Educational attainment**										
Yr 12 or above (7975)	50.29	49.92	85.59	81.92	75.86	71.22	61.46	82.40	83.43	74.17
Yr 11 or less (4041)	47.15	49.56	76.64	73.48	70.22	66.32	59.31	78.82	78.74	72.00
*F(1.475)*	*159.19*	*2.48*	*235.31*	*112.92*	*95.55*	*102.68*	*24.48*	*42.39*	*40.34*	*28.45*
	***	*ns*	***	***	***	***	***	***	***	***
**Employment status**										
Employed (7741)	51.77	50.25	89.04	87.14	78.54	73.40	62.57	85.13	86.53	74.87
Unemployed (520)	51.22	47.08	85.07	82.37	76.28	70.67	62.85	77.57	76.39	68.72
*F(1.471)*	*1.58*	*36.90*	*17.16*	*10.00*	*3.69*	*6.47*	*0.10*	*46.04*	*36.74*	*44.94*
	*ns*	***	***	**	*ns*	*	*ns*	***	***	***
**Housing status**										
Own/mortgage (9281)	49.12	50.43	82.58	78.86	74.20	69.99	61.16	82.54	83.10	74.48
Rent (3039)	49.53	47.97	82.24	79.63	73.01	68.26	59.46	77.15	78.18	70.38
*F(1.475)*	*1.77*	*92.54*	*0.20*	*0.75*	*2.79*	*6.35*	*11.43*	*72.65*	*36.32*	*81.86*
	*ns*	***	*ns*	*ns*	*ns*	*	***	***	***	***
**Receipt of welfare payments (working age respondents)**										
Non-recipients (8373)	51.75	50.16	88.93	86.86	78.45	73.25	62.73	84.87	86.09	74.66
Welfare recipients (2091)	46.57	46.16	76.22	68.35	66.22	62.71	55.48	70.35	71.10	66.50
*F(1,473)*	*259.15*	*174.85*	*305.32*	*300.00*	*223.36*	*274.19*	*147.06*	*431.71*	*241.05*	*221.56*
	***	***	***	***	***	***	***	***	***	***

Degrees of freedom for *F *statistics based on number of clusters (primary sampling units) and number of strata

* *p *< .05, ** *p *< .01, *** *p *< .001

Age effects were also consistent with expectations. While those in older age groups reported poorer levels of health across most SF-36 scales, the decline was most pronounced in the measures related to physical health (Physical Health, Bodily Pain). In contrast to this pattern, however, both the Mental Health scale and Mental Component Summary Score showed improved mental health with increasing age, apart from a slight decline evident in the oldest (75+) age group. This is consistent with epidemiological survey results using a variety of mental health measures [27]. Health differences associated with current marital status (comparing partnered respondents with those who were separated or divorced) were evident across all scales. The greatest differences were observed in the Social Functioning and Role Emotional scales.

The analysis showed health was associated with a range of measures of social status, however the profile across the SF-36 scales differed for the different measures. Consideration of educational attainment showed that those respondents who had less than secondary education had significantly poorer physical health, most evident on the Physical Functioning scale. Current unemployment (compared to full or part-time employment) was significantly associated with poorer health on a number of scales, but particularly the measures loading on the mental health factor (Social Functioning, Role Emotional and Mental Health). This too, is consistent with the extensive unemployment literature [28]. Housing tenure (rental housing vs other) was not related to the SF-36 physical health scales. However, reliance on rental housing was associated with poorer mental health (lower means on Vitality, Social Functioning, Role Emotional, and Mental Health). While Australian welfare payments are universally available, eligibility is highly targeted. Therefore, welfare receipt appears to be a good proxy for poor financial circumstances. In addition, a subset of the welfare population are those with severe disabilities that prevent work. Consistent with this, we found that, amongst working age respondents, receipt of an Australian income support payment was strongly associated with both poorer physical and mental health.

The final set of comparisons examined the association between SF-36 scales and respondents' health and social circumstances as measured by other scales and items included in the HILDA Survey (Table 6). Consistent with expectations, respondents who reported a long-term health condition or disability demonstrated lower mean scores on all SF-36 scales, but particularly so on the scales loading on physical health (Physical Functioning, Role Physical, Bodily Pain and General Health). A similar pattern of associations was observed when examining the circumstances of those with health conditions that impacted on their ability to work. The greater influence on work ability was associated with poorest physical health as measured by SF-36 scales. Reported satisfaction with health was most strongly associated with General Health and physical health measures more generally, while overall satisfaction with one's life was more highly associated with the SF-36 scales related to mental health.

**Table 6:** Mean SF-36 Scale and Component Summary Scores, by other measures of health and social situation. The *F *statistic associated with each comparison is indicated, together with minimum number of respondents in each cell (in brackets).

	**PCS**	**MCS**	**PF**	**RP**	**BP**	**GH**	**VT**	**SF**	**RE**	**MH**
**Presence of long term health condition or disability**										
No (9549)	52.16	50.58	88.57	88.09	80.14	74.85	64.19	85.87	86.55	75.34
Yes (2771)	38.89	47.02	61.99	47.91	52.96	51.40	49.10	65.35	65.48	66.94
*F(1,475)*	*1797.99*	*163.21*	*1324.59*	*1367.80*	*1621.36*	*1777.15*	*892.23*	*858.84*	*455.68*	*340.62*
	***	***	***	***	***	***	***	***	***	***
**If so, does condition limit ability to work?**										
No (761)	47.69	49.20	80.13	75.08	70.20	65.01	58.66	79.67	78.80	72.31
Yes (1969)	35.76	46.23	56.05	38.30	46.92	46.57	45.72	60.36	60.98	65.08
Can do nothing (41)	28.00	45.10	31.05	22.33	37.13	37.61	41.20	51.72	44.01	61.01
*F(2,460)*	*475.53*	*15.92*	*361.10*	*275.57*	*272.20*	*235.87*	*110.07*	*159.16*	*65.12*	*35.12*
	***	***	***	***	***	***	***	***	***	***
**Satisfaction with health**										
Satisfied/mixed (11182)	50.22	50.40	84.65	82.24	76.19	71.88	62.43	83.26	84.14	74.65
Not satisfied (1018)	39.71	43.95	62.67	48.61	52.82	47.63	45.23	61.96	58.97	62.24
*F(1,475)*	*501.04*	*204.52*	*391.67*	*477.58*	*516.14*	*672.21*	*503.83*	*435.33*	*250.56*	*270.29*
	***	***	***	***	***	***	***	***	***	***
**Satisfaction with life**										
Satisfied/mixed (11937)	49.40	50.22	82.94	79.90	74.51	70.23	61.42	82.07	82.87	74.21
Dissatisfied (341)	43.99	37.17	69.34	53.93	56.02	49.22	40.10	53.42	51.37	50.20
*F(1,475)*	*48.84*	*265.85*	*64.26*	*84.88*	*98.41*	*196.47*	*275.57*	*257.92*	*125.10*	*364.73*
	***	***	***	***	***	***	***	***	***	***
**Report that job stress makes physically ill**										
Not agree, neither (6533)	51.82	51.12	89.11	87.80	79.22	74.59	64.24	86.25	88.23	76.08
Agree (1255)	49.89	44.72	84.90	76.61	71.08	65.19	53.41	74.52	70.55	66.04
*F(1,472)*	*40.41*	*290.07*	*39.02*	*98.10*	*89.70*	*174.22*	*267.47*	*208.13*	*200.02*	*269.57*
	***	***	***	***	***	***	***	***	***	***
**Often Lonely**										
Disagree/mixed (9506)	49.74	51.33	84.16	81.58	75.93	71.65	63.04	84.27	85.63	76.17
Agree (2454)	47.45	43.87	77.37	69.90	66.63	61.96	52.26	70.07	67.19	63.14
*F(1,475)*	*63.52*	*722.01*	*100.92*	*147.99*	*196.06*	*315.39*	*486.49*	*514.90*	*424.96*	*763.49*
	***	***	***	***	***	***	***	***	***	***

Degrees of freedom for *F *statistics based on number of clusters (primary sampling units) and number of strata

* *p *< .05, ** *p *< .01, *** *p *< .001

The final two criterion measures (job stress and loneliness) were expected to be most strongly associated with poorer mental health outcomes. Consistent with expectations, those respondents in work who reported that their experience of job stress made them physically ill reported poorest health on the Vitality and Mental Health scales, while those who reported often feeling lonely had low scores on the Mental Health, Social Functioning and Vitality scales.

## Conclusions

This analysis has demonstrated the validity of the SF-36 data collected through the first wave of the HILDA Survey. The eight scales were shown to be psychometrically sound, with good internal consistency, discriminant validity and high reliability. The results supported the underlying two-factor structure, with factors related to physical and mental health. The pattern of factor loadings, variance explained, and the profile and magnitude of the scale means were consistent with previous Australian and international results. There was, however, an indication that the estimated population means on several of the SF-36 scales, particularly on scales loading on the mental health factor, were lower in the HILDA Survey than previous Australian national data collected through the National Health Survey. Self-report measures of health status can be subject to a range of biases, such as reporting bias or cohort effects. They may also be influenced by contextual factors, such as the setting in which the measure is conducted. Further consideration of these different data sources, and an examination of possible methodological and other differences, is warranted.

With respect to the relationship between SF-36 scales and external criteria, we demonstrated a pattern of results consistent with expectations and previous research. We examined measures having differential effects on physical and mental health (e.g., disability and health satisfaction vs job stress, loneliness, and life satisfaction). The relationship between demographic characteristics (age, sex, marital status) and SF-36 scales was consistent with previous data. Finally, we focused on health inequalities, using a range of markers of social status and examining their relationship with the SF-36 scales. While each marker of social status was associated with poorer health, the dimensions of health related to each social variable differed. Whereas educational attainment was most strongly associated with poorer physical health, housing tenure and employment status were related to mental health scales. The measure of welfare receipt, which is associated with economic hardship, was strongly associated with both poorer physical and mental health.

The application of the probability weights available with the HILDA Survey data ensures the accuracy of the point-estimates of the population parameters, such as the mean, by correcting for non-response and design characteristics. However, it is also important to recognise the impact of the survey design (clustering and stratification) on the calculation of standard errors. As the sampling units in the HILDA Survey are Census Collection Districts and, within that, households, the data contradict assumptions of independence and should not be treated as a simple random sample drawn from the population. We expect more similarity between individuals within Census Collection Districts and households than between those drawn at random from the population and if these characteristics of the survey design are not taken into account the estimates of standard error will overstate accuracy.

Calculation of the design effect provides an indication of the increase in error associated with the complex survey design, representing the ratio of the variance for the complex design to that obtained assuming a simple random sample. These measures range from 1.4 (for Role Emotional scale) to 3.1 (for the Physical Functioning scale). Thus, whereas the 95 percent confidence interval around the population estimate for Physical Functioning Scale (84.49) would have been 82.04 to 82.94 had we not taken the survey design into account, our estimate was 81.77 to 83.21. The design effects were greater for the scales assessing physical health than for those loading on mental health. This suggests physical health more strongly clusters within households and areas than does mental health, perhaps as a consequence of the clustering of age (design effect of 3.8) and the strong relationship between age and physical health and disability. Note that the design effects are not evident in the population estimates in Table 4 as these figures were corrected to provide an estimate of the population mean and population standard deviation. Nonetheless, the design effects highlight the need to utilise appropriate statistical techniques in analysis of the HILDA Survey data.

This analysis has provided evidence that the SF-36 data collected in the HILDA Survey is valid and supports its use as a general outcome measure of physical and mental health status. It also suggests that the results obtained using the SF-36 in the HILDA Survey can be interpreted by reference to published SF-36 normative data and comparison with previous research findings. Most critically, from our perspective, the analysis provides initial support for the proposition that the SF-36 scales and component summary scores are related in a meaningful and interpretable manner to measures of social status. This supports the use of the HILDA Survey data in our analysis of health inequalities and the effects of social exclusion in Australia.

## Competing interests

The authors declare that they have no competing interests.

## Authors' contributions

PB and TC participated in all aspects of the preparation of this manuscript, including data analysis, preparation of various sections of the manuscript, and commenting on drafts.

## Pre-publication history

The pre-publication history for this paper can be accessed here:



## Supplementary Material

Additional File 1 Additional file SF36 HILDA validation.pdf – provides tables and figures used to determine item-discriminant validity and to explore the pattern of missing data across SF-36 items. Click here for file
