# Supplementary material for: The validity of the SF-36 in an Australian National Household Survey: demonstrating the applicability of the Household Income and Labour Dynamics in Australia (HILDA) Survey to examination of health inequalities
Source: BMC Public Health. 2004 Oct 7;4:44. doi: 10.1186/1471-2458-4-44 (PMC524495; doi:10.1186/1471-2458-4-44)
Supplement: Additional File 1 — Additional file SF36 HILDA validation.pdf – provides tables and figures used to determine item-discriminant validity and to explore the pattern of missing data across SF-36 items. [file 1471-2458-4-44-S1.rtf]

Table A.1:  Item descriptive statistics and Pearson item-scale correlations corrected for overlap.
Item			Pearson item-scale correlations*	
Name	Label	Mean	SD	PF	RP	BP	GH	VT	SF	RE	MH	
Scale = PF (Physical Functioning)											
PF01	Vigorous	1.31	2.76	0.55*	0.49	0.47	0.49	0.33	0.32	0.23	0.16	
PF02	Moderate	1.83	2.83	0.81*	0.55	0.51	0.47	0.35	0.43	0.29	0.22	
PF03	Lifting	1.87	2.84	0.78*	0.51	0.48	0.44	0.33	0.43	0.29	0.23	
PF04	Climbsev	1.69	2.84	0.77*	0.51	0.46	0.49	0.36	0.38	0.27	0.20	
PF05	Climbone	1.87	2.90	0.81*	0.46	0.41	0.40	0.29	0.36	0.26	0.19	
PF06	Bending	1.72	2.83	0.76*	0.49	0.51	0.43	0.32	0.36	0.26	0.19	
PF07	Walk-km	1.79	2.85	0.81*	0.51	0.46	0.46	0.34	0.40	0.27	0.21	
PF08	Walk1/2km	1.88	2.89	0.81*	0.44	0.40	0.39	0.29	0.37	0.25	0.19	
PF09	Walkone	1.93	2.91	0.70*	0.32	0.29	0.28	0.21	0.30	0.21	0.15	
PF10	Bathing	2.01	2.86	0.58*	0.25	0.23	0.22	0.16	0.26	0.18	0.14	
Scale = RP (Role Physical)											
RP01	Cutdown	1.07	2.57	0.48	0.76*	0.53	0.43	0.38	0.51	0.40	0.24	
RP02	Accomplished	0.98	2.56	0.49	0.77*	0.54	0.47	0.43	0.51	0.43	0.29	
RP03	Limited	1.01	2.58	0.56	0.81*	0.57	0.48	0.39	0.50	0.37	0.24	
RP04	Difficult	0.99	2.57	0.54	0.79*	0.60	0.50	0.44	0.52	0.41	0.28	
Scale = BP (Bodily Pain) 											
BP01	Magnitude	1.65	2.90	0.50	0.57	0.80*	0.53	0.47	0.51	0.32	0.33	
BP02	Interfere	1.07	2.65	0.57	0.67	0.80*	0.56	0.49	0.61	0.40	0.37	
Scale = GH (General Health)											
GH01	General	1.77	2.84	0.52	0.50	0.53	0.70*	0.51	0.45	0.33	0.35	
GH02	Easier	3.21	3.37	0.28	0.32	0.35	0.49*	0.41	0.42	0.31	0.40	
GH03	Healthy	1.46	2.84	0.37	0.38	0.40	0.66*	0.47	0.41	0.28	0.36	
GH04	Worse	2.66	3.26	0.35	0.32	0.36	0.47*	0.36	0.28	0.22	0.27	
GH05	Excellent	1.54	2.93	0.48	0.49	0.52	0.77*	0.57	0.49	0.35	0.42	
Scale = VT (Vitality)											
VT01	Life	2.14	3.02	0.35	0.42	0.44	0.55	0.64*	0.54	0.42	0.58	
VT02	Energy	2.31	3.10	0.39	0.45	0.46	0.59	0.67*	0.49	0.37	0.53	
VT03	Worn	3.35	3.37	0.25	0.32	0.36	0.41	0.63*	0.45	0.33	0.52	
VT04	Tired	3.05	3.24	0.24	0.31	0.37	0.42	0.66*	0.44	0.33	0.49	
Scale = SF (Social Functioning)											
SF01	Extent	1.04	2.64	0.43	0.56	0.54	0.49	0.53	0.71*	0.60	0.55	
SF02	Time	3.37	3.24	0.42	0.51	0.51	0.50	0.56	0.71*	0.52	0.58	
Scale = RE (Role Emotional)											
RE01	Cutdown	1.08	2.57	0.30	0.44	0.34	0.35	0.39	0.54	0.72*	0.45	
RE02	Accomplished	1.00	2.55	0.27	0.40	0.32	0.34	0.41	0.54	0.72*	0.47	
RE03	Not careful	1.05	2.59	0.28	0.37	0.31	0.34	0.37	0.50	0.68*	0.44	
Scale = MH (Mental Health)											
MH01	Nervous	3.97	3.49	0.17	0.18	0.22	0.30	0.36	0.39	0.34	0.54*	
MH02	Nocheer	4.28	3.57	0.23	0.25	0.29	0.36	0.46	0.53	0.46	0.66*	
MH03	Calm	2.19	3.07	0.16	0.23	0.29	0.38	0.57	0.43	0.37	0.58*	
MH04	Down	3.82	3.45	0.21	0.27	0.32	0.38	0.56	0.54	0.47	0.69*	
MH05	Happy	1.77	2.87	0.15	0.23	0.27	0.38	0.54	0.45	0.37	0.60*	
*  Item-scale correlation corrected for overlap (relevant item removed from its scale for correlation).  Starred correlations also hypothesized to be highest in the same row.
Abbreviations: PF = Physical Functioning; RP = Role Physical; BP = Bodily Pain; GH = General Health; VT = Vitality; SF = Social Functioning; RE = Role Emotional; MH = Mental Health. 


Table A.2: Item-level discriminant validity tests.

Item				
Name	Label	Mean	SD	PF	RP	BP	GH	VT	SF	RE	MH	
Scale = PF (Physical Functioning)											
PF01	Vigorous	1.31	2.76	**	2	2	2	2	2	2	2	
PF02	Moderate	1.83	2.83	**	2	2	2	2	2	2	2	
PF03	Lifting	1.87	2.84	**	2	2	2	2	2	2	2	
PF04	Climbsev	1.69	2.84	**	2	2	2	2	2	2	2	
PF05	Climbone	1.87	2.90	**	2	2	2	2	2	2	2	
PF06	Bending	1.72	2.83	**	2	2	2	2	2	2	2	
PF07	Walk-km	1.79	2.85	**	2	2	2	2	2	2	2	
PF08	Walk1/2km	1.88	2.89	**	2	2	2	2	2	2	2	
PF09	Walkone	1.93	2.91	**	2	2	2	2	2	2	2	
PF10	Bathing	2.01	2.86	**	2	2	2	2	2	2	2	
Scale = RP (Role Physical)											
RP01	Cutdown	1.07	2.57	2	**	2	2	2	2	2	2	
RP02	Accomplished	0.98	2.56	2	**	2	2	2	2	2	2	
RP03	Limited	1.01	2.58	2	**	2	2	2	2	2	2	
RP04	Difficult	0.99	2.57	2	**	2	2	2	2	2	2	
Scale = BP (Bodily Pain) 											
BP01	Magnitude	1.65	2.90	2	2	**	2	2	2	2	2	
BP02	Interfere	1.07	2.65	2	2	**	2	2	2	2	2	
Scale = GH (General Health)											
GH01	General	1.77	2.84	2	2	2	**	2	2	2	2	
GH02	Easier	3.21	3.37	2	2	2	**	2	2	2	2	
GH03	Healthy	1.46	2.84	2	2	2	**	2	2	2	2	
GH04	Worse	2.66	3.26	2	2	2	**	2	2	2	2	
GH05	Excellent	1.54	2.93	2	2	2	**	2	2	2	2	
Scale = VT (Vitality)											
VT01	Life	2.14	3.02	2	2	2	2	**	2	2	2	
VT02	Energy	2.31	3.10	2	2	2	2	**	2	2	2	
VT03	Worn	3.35	3.37	2	2	2	2	**	2	2	2	
VT04	Tired	3.05	3.24	2	2	2	2	**	2	2	2	
Scale = SF (Social Functioning)											
SF01	Extent	1.04	2.64	2	2	2	2	2	**	2	2	
SF02	Time	3.37	3.24	2	2	2	2	2	**	2	2	
Scale = RE (Role Emotional)											
RE01	Cutdown	1.08	2.57	2	2	2	2	2	2	**	2	
RE02	Accomplished	1.00	2.55	2	2	2	2	2	2	**	2	
RE03	Not careful	1.05	2.59	2	2	2	2	2	2	**	2	
Scale = MH (Mental Health)											
MH01	Nervous	3.97	3.49	2	2	2	2	2	2	2	**	
MH02	Nocheer	4.28	3.57	2	2	2	2	2	2	2	**	
MH03	Calm	2.19	3.07	2	2	2	2	1	2	2	**	
MH04	Down	3.82	3.45	2	2	2	2	2	2	2	**	
MH05	Happy	1.77	2.87	2	2	2	2	2	2	2	**	
** Discriminant validity test not conducted.
Cutoff point for significance is 2 standard errors.
Levels of scaling success:
2 – Item-scale correlation is significantly higher for hypothesized scale than for competing scale;
1 – Item-scale correlation is higher for hypothesized scale than competing scale, but not significantly;
-1 – Item-scale correlation is lower for hypothesized scale than competing scale, but not   significantly;
-2 – Item-scale correlation is significantly lower for hypothesized scale than for competing scale.
Abbreviations: PF = Physical Functioning; RP = Role Physical; BP = Bodily Pain; GH = General Health; VT = Vitality; SF = Social Functioning; RE = Role Emotional; MH = Mental Health.


Table A.3:  Frequency and percentage of item-scale correlations at each level of scaling success.

	-2		-1		1		2		1 + 2	
Scale	n	%		n	%		n	%		n	%		n	%	
PF	0	0.0		0	0.0		0	0.0		70	100.0		70	100.0	
RP	0	0.0		0	0.0		0	0.0		28	100.0		28	100.0	
BP	0	0.0		0	0.0		0	0.0		14	100.0		14	100.0	
GH	0	0.0		0	0.0		0	0.0		35	100.0		35	100.0	
VT	0	0.0		0	0.0		0	0.0		28	100.0		28	100.0	
SF	0	0.0		0	0.0		0	0.0		14	100.0		14	100.0	
RE	0	0.0		0	0.0		0	0.0		21	100.0		21	100.0	
MH	0	0.0		0	0.0		1	2.9		34	97.1		35	100.0	
Levels of scaling success:
2 – Item-scale correlation is significantly higher for hypothesized scale than for competing scale;
1 – Item-scale correlation is higher for hypothesized scale than competing scale, but not significantly;
-1 – Item-scale correlation is lower for hypothesized scale than competing scale, but not   significantly;
-2 – Item-scale correlation is significantly lower for hypothesized scale than for competing scale.
Abbreviations: PF = Physical Functioning; RP = Role Physical; BP = Bodily Pain; GH = General Health; VT = Vitality; SF = Social Functioning; RE = Role Emotional; MH = Mental Health.

Figure A.1:  Mean percent of non-responses/missing values to individual items of SF-36 (item number presented above), grouped by scale.  
